# Stimulated by Novelty? The Role of Psychological Needs and Perceived Creativity

**DOI:** 10.1177/0146167217752361

**Published:** 2018-02-06

**Authors:** Kiki M. M. De Jonge, Eric F. Rietzschel, Nico W. Van Yperen

**Affiliations:** 1 University of Groningen, The Netherlands

**Keywords:** brainstorming, cognitive stimulation, creativity, need for structure, need for autonomy

## Abstract

In the current research, we aimed to address the inconsistent finding in the brainstorming literature that cognitive stimulation sometimes results from novel input, yet other times from non-novel input. We expected and found, in three experiments, that the strength and valence of this relationship are moderated by people’s psychological needs for structure and autonomy. Specifically, the effect of novel input (vs. non-novel input), through perceived creativity, on cognitive stimulation was stronger for people who were either low in need for structure or high in need for autonomy. Also, when the input people received did not fit their needs, they experienced less psychological cognitive stimulation from this input (i.e., less task enjoyment and feeling more blocked) compared with when they did not receive any input. Hence, to create the ideal circumstances for people to achieve cognitive stimulation when brainstorming, input novelty should be aligned with their psychological needs.

People often work together on a variety of tasks, including idea generation in brainstorming sessions (Chirumbolo, Mannetti, Pierro, Areni, & Kruglanski, 2005; Nijstad, Stroebe, & Lodewijkx, 2006). In brainstorming groups, members contribute different knowledge, expertise, and opinions. Receiving input from others can be cognitively stimulating and result in more and better ideas than individual idea generation (Paulus & Coskun, 2013), but can also be interfering and interrupt one’s own thought process, hence resulting in suboptimal performance (Diehl & Stroebe, 1987; Nijstad & Stroebe, 2006).

The degree to which sharing of ideas results in cognitive stimulation depends on factors such as the attention given to these ideas and the type of ideas shared, including their semantic diversity and novelty (Dugosh & Paulus, 2005; Nijstad, Stroebe, & Lodewijkx, 2002). So far, research focusing on input novelty has found inconsistent results, sometimes indicating that novel input rather than non-novel input increases cognitive stimulation (e.g., Berg, 2014), other times indicating the opposite (e.g., Dugosh & Paulus, 2005). The present research adds to the literature by arguing and demonstrating that the strength and valence of the link between input novelty and cognitive stimulation partly depends on people’s psychological needs for structure and autonomy. In addition, we propose that the perceived creativity of the input mediates this relationship, in line with previous research indicating that the role of novelty in the perception of creativity is less than straightforward (e.g., Mueller, Wakslak, & Krishnan, 2014). Moreover, we extend the definition and measurement of cognitive stimulation by including both performance and psychological factors as components. We discuss this below.

## Cognitive Stimulation in Group Brainstorming

Usually, brainstorming groups perform below their potential as a result of production blocking (Diehl & Stroebe, 1987; Lamm & Trommsdorff, 1973; Nijstad & Stroebe, 2006). Being exposed to other group members’ ideas can interfere with one’s own idea generation process, simply because one typically has to wait for another group member to stop talking before being able to contribute one’s own idea. Furthermore, monitoring others’ input may lead to cognitive interference, resulting in less effective idea generation (Diehl & Stroebe, 1991). Nevertheless, one important reason for working together on brainstorming tasks is the potential for *cognitive stimulation*: Being exposed to other people’s ideas might enhance one’s own idea generation process (e.g., Nijstad & Stroebe, 2006).

Previous research has focused on *performance components* of cognitive stimulation, such as *productivity* and *idea diversity*. When people are exposed to other people’s ideas, the features of the input are used to increase *productivity* by generating new ideas through combining knowledge and forming new associations. Indeed, previous findings indicate that when group members exchange and collectively process information, the group has the potential, at least in theory, to perform better than the sum of its parts (i.e., all individuals separately) (e.g., De Dreu, Nijstad, & van Knippenberg, 2008; Hinsz, Tindale, & Vollrath, 1997). Group brainstorming may increase *idea diversity* because group members can contribute different knowledge, expertise, and opinions to the group, which may trigger new ideas or areas of knowledge in one’s own mind that would not be as easily activated without some external cue (Brown, Tomeo, Larey, & Paulus, 1998; Dugosh, Paulus, Roland, & Yang, 2000; Nijstad & Stroebe, 2006).

We extend the definition of cognitive stimulation by suggesting that it also entails *psychological components*, namely, *task enjoyment* and *reduced feelings of being blocked*. We expect high levels of *task enjoyment*, because the feeling of being able to use others’ ideas is likely to be valued positively and increase intrinsic motivation (Amabile, 1983). Also, *reduced feelings of being blocked* by the input are expected because input that is cognitively stimulating is likely to be perceived as helpful for idea generation. Indeed, previous research indicates that people are generally more satisfied and perceive idea generation as easier when brainstorming in groups compared with brainstorming individually (Nijstad & Stroebe, 2006; Nijstad et al., 2006). An important factor explaining this finding is the feeling that group brainstorming results in fewer failures to generate ideas, as the group together is able to continue generating input even at moments when the individual is unable to come up with an idea. Furthermore, people tend to believe that group brainstorming is very effective, because they ascribe the reduction of failures to the stimulating effect of receiving other people’s ideas (Nijstad et al., 2006). Three variables that may explain whether others’ input cognitively stimulates rather than interferes are input novelty, the individual’s psychological needs, and perceived creativity.

## Input Novelty and Cognitive Stimulation

The extent to which cognitive stimulation occurs partly depends on characteristics of the input ideas, including idea novelty (Dugosh & Paulus, 2005). Although one might intuitively expect that idea novelty enhances cognitive stimulation (see, for example, Connolly, Routhieaux, & Schneider, 1993), its role appears to be complex: Some findings suggest that novelty increases cognitive stimulation, whereas other findings suggest the opposite.

Kohn, Paulus, and Choi (2011) found that brainstorming groups were more likely to come up with novel combinations of ideas when they had been presented with rare (as opposed to common) ideas. Also, findings by Berg (2014) indicate that exposing people to new ideas stimulates the production of novel ideas. In addition, Agogué and colleagues (2013) found that presenting people with unusual (as opposed to common) solutions improved original problem solving. They argue that presenting common solutions results in a fixation on common knowledge and hence in usual rather than novel solutions. This fixation effect is in line with findings by Perttula and Sipilä (2007), who found that use of common examples (as opposed to novel examples) when brainstorming causes more fixation and results in reproduction of features of the examples presented.

In contrast to these findings, Dugosh and Paulus (2005) found that participants’ productivity in a brainstorming task was stimulated most when participants were presented with a large number of highly common, conventional ideas as opposed to unique, novel ideas. They argue that common ideas are likely to be closely related to one’s own mental images, creating the greatest opportunity to elicit ideas associated with the input. In addition, Connolly and colleagues (1993) found (in contrast to their expectation) that common rather than rare input stimulated more novel idea generation. Kohn and Smith (2011) found that participants exposed to a low number of common categories generated more novel ideas than those exposed to novel categories. Finally, Fink and colleagues (2010) found that people generated more original responses when receiving common rather than nonsense input, but found no stimulation effect of novel input. They suggest that novel input is highly complex to process and makes it difficult for participants to keep up with the generation of ideas at the same level of those presented.

**Figure 1.**
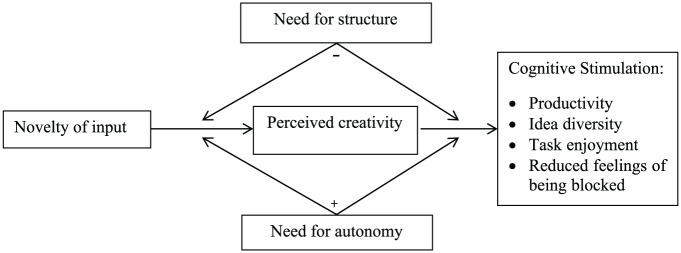
Theoretical model. *Note.* Cognitive stimulation as an indirect function of input novelty, mediated by perceived creativity, and moderated by need strengths (i.e., need for structure and need for autonomy).

The complex role of input novelty in the creative process raises the question of which factors could affect whether or not novel input during brainstorming is perceived as creative (cf. Zhou, Wang, Song, & Wu, 2017) and hence leads to cognitive stimulation. We extend the literature by indicating that the (mis)fit between input novelty and the individual’s psychological needs moderates this link (see also Figure 1 for our theoretical model). Individual needs are important predictors and moderators in the context of creative performance, work motivation, and group interactions (e.g., Chirumbolo, Livi, Mannetti, Pierro, & Kruglanski, 2004; Deci & Ryan, 2000; Van Yperen, Wörtler, & De Jonge, 2016). In the current research, we focused on need for structure and need for autonomy, because these independent needs form a dynamic duo, often relating to opposing outcomes within the same context. For example, autonomous situations characterized by freedom fit well with the need for autonomy, but are not beneficial for those high in need for structure, as such situations often imply a lack of structure (Rietzschel, 2015; Slijkhuis, Rietzschel, & Van Yperen, 2013). In fact, people high in need for structure prefer a predetermined task structure over high autonomy (Rietzschel, Slijkhuis, & Van Yperen, 2014). Moreover, findings on group performance and group creativity suggest that the way people attend to and make use of others’ input depends on both epistemic (such as need for structure) and social motives (such as need for autonomy) (De Dreu et al., 2008). As explained below, different levels of cognitive stimulation are to be expected for these psychological needs when people receive input high versus low in novelty, with novel input being less beneficial for those high in need for structure as well as for those low in need for autonomy.

## Cognitive Stimulation and Need for Structure

People high in need for structure have a strong preference for clarity and predictability, an aversion to extensive information processing, and a strong desire to diminish ambiguity and uncertainty (Neuberg & Newsom, 1993; Thompson, Naccarato, Parker, & Moskowitz, 2001). They perform worse in ambiguous task conditions and tend to experience high levels of stress and discomfort when confronted with ill-structured situations that lack clarity (Beersma, De Dreu, Dalenberg, & Vogelaar, 2007). Hence, they tend to form and use simple cognitive structures (such as cognitive heuristics and schemas) with the aim of simplifying the environment into a manageable form (Neuberg & Newsom, 1993). Moreover, people with a high need for structure perform most creatively under conditions of clarity and focus (Rietzschel, De Dreu, & Nijstad, 2007; Rietzschel et al., 2014).

Research on need for structure and related epistemic needs (such as need for closure and uncertainty avoidance) suggests that such people will not respond very favorably to novel input during brainstorming. Novel input is surprising and forms a schema violation of one’s own activated cognitive structures (Gocłowska, Baas, Crisp, & De Dreu, 2014). Also, it can make the task more complex and ambiguous (Fink et al., 2010) and requires more information processing (Förster, 2009). These aspects are disliked by those high in need for structure and make it difficult to understand and incorporate the input when brainstorming. Novel input is therefore likely to disrupt the idea generation process (Nijstad & Stroebe, 2006) and hence to lead to a sense of being blocked. Resulting from this, people high in need for structure are expected to experience high levels of being blocked when receiving novel input. Also, such schema violations impede their creative performance (Gocłowska et al., 2014).

All in all, people high in need for structure are not likely to value novel input as helpful or creative, and, as a result, are expected not to be particularly stimulated by it. Rather, these people are likely to respond more positively to less original ideas. Because non-novel input is easily recognized as highly relevant to the task and is more likely to resemble the ideas that the person has been generating (Dugosh & Paulus, 2005), it may reaffirm the task goal, thus increasing task clarity and lowering ambiguity. People with a high need for structure, who dislike the ambiguity and complexity associated with highly original ideas, may also be more motivated to attend to less original ideas, which is also an important precondition for cognitive stimulation effects (Dugosh et al., 2000). Common ideas may seem more valid (Stasser & Birchmeier, 2003) and result in the least cognitive resistance (Berg, 2014)—heuristics that people with a high need for structure may be especially likely to use. As common ideas are likely to be closely related to one’s own semantic schemas, such input creates the greatest opportunity to elicit ideas associated with the input (Dugosh & Paulus, 2005). Given that people with a high need for structure prefer clarity, predictability, and certainty, non-novel input should fit their cognitive needs better than novel input. Thus, we expected that the effect of novel (vs. non-novel) input on cognitive stimulation would be stronger (vs. weaker) for people with a low need for structure than for people with a higher need for structure.

## Cognitive Stimulation and Need for Autonomy

Besides the epistemic implications of the input one receives, the mere fact that ideas are shared and need to be attended to may be problematic for some people, especially those who desire freedom, independence, and individual discretion. Such people, who are high in *need for autonomy* (Deci & Ryan, 2000), prefer to be in control of their own actions and to decide on their own how and when to perform a task (Hackman & Oldham, 1976). They prefer task outcomes to depend on their own decisions, initiatives, and efforts; they dislike external instructions; and they show an aversion to external control (Van den Broeck, Vansteenkiste, De Witte, & Lens, 2008).

When brainstorming, people high in need for autonomy will probably perceive external input as controlling and interrupting their workflow, particularly when the ideas received are non-novel. The forced delay of having to attend to other people’s ideas is an important component of production blocking (Diehl & Stroebe, 1987; Nijstad & Stroebe, 2006), and it is likely that this is especially annoying when receiving common input that does not seem to add anything new. This kind of non-novel input is likely to be perceived as having no added value for executing the task at hand (that is, the idea does not add anything that one could not have generated oneself), leaving only an unnecessary interruption and a form of external control. Such external control violates their need for autonomy, and this is known to lower intrinsic motivation and creativity (see, for example, Shalley, Zhou, & Oldham, 2004).

In contrast, receiving novel input is expected to attenuate these negative effects, because novel input adds a new and original perspective to the task at hand. Novel input may enhance people’s flexibility and freedom in approaching the task, because it gives them more options to choose from. This line of reasoning fits with previous findings indicating that external control and constraints undermine creative performance, whereas intrinsic motivation (fueled by perceived autonomy) enhances creativity (e.g., Amabile, 1983). Thus, the effect of novel (vs. non-novel) input on cognitive stimulation should be stronger for people with a high need for autonomy than for people with a low need for autonomy.

## The Role of Perceived Creativity

Besides addressing important moderators on the relation between input novelty and cognitive stimulation, we argue that these effects will be mediated by the *perceived creativity* of the input (see Figure 1). Although recognizing creativity revolves around the perception of other people’s ideas, whereas cognitive stimulation concerns generating ideas oneself, the two processes are strongly interrelated. For example, Zhou et al. (2017) argue that the recognition of an idea’s novelty is crucial for its potential to further stimulate the creative process, and previous research indicates that recognizing creativity is linked to the stimulating potential of the input (Gawronski & Bodenhausen, 2006; cf. Zhou et al., 2017). Thus, the degree to which an idea activates associations that can stimulate idea generation is a function of the degree to which the idea is appreciated and seen as creative. Also, ideas that score high on richness, in the sense of triggering further idea generation, are perceived as more creative (Sosa & Dong, 2013). Based on this, we argue that input is perceived as more creative when it activates a higher amount of task-relevant associations in one’s mind. This in turn should result in higher levels of cognitive stimulation. Hence, recognizing the creativity and the added value of input is an important first step in the cognitive stimulation process.

Yet, although generating and recognizing novelty clearly is at the heart of the creative process (Diedrich, Benedek, Jauk, & Neubauer, 2015; Runco & Charles, 1993; Zhou et al., 2017), people do not always respond favorably to novel ideas. They sometimes do not recognize (Mueller et al., 2014) or appreciate creativity. In fact, people may have an implicit bias against creativity, even when they explicitly claim to find it valuable (Mueller, Melwani, & Goncalo, 2012). Other research also shows considerable variability in people’s recognition of creative ideas (e.g., Herman & Reiter-Palmon, 2011; Silvia, 2008). In line with the previously discussed research, we anticipated that different psychological needs would result in different perceptions of the creativity of novel input.

First, we expected that people high in need for autonomy would perceive novel ideas as more creative. Because novel input is surprising and can stimulate remote associations, and hence could help them generate new ideas (Kohn et al., 2011), they may be especially likely to perceive original and unusual input as a useful contribution to their own idea generation (see, for example, Connolly et al., 1993). Hence, novel ideas should be appreciated as creative input by people high in need for autonomy, and this in turn should result in higher cognitive stimulation than non-novel input.

Second, people high in need for structure will probably *not* appreciate novel input as creative, precisely because novel ideas are surprising and add a new perspective. For example, people high in need for closure are less open to new or novel input when brainstorming (as well as in other group tasks), and hence generate fewer (creative) ideas (Chirumbolo et al., 2004; Chirumbolo et al., 2005; De Dreu et al., 2008). Moreover, people who have a proximal, concrete processing style, or who have a high motivation to reduce uncertainty, tend to evaluate creative ideas more negatively (Mueller et al., 2012; Mueller et al., 2014). Also, people who are oriented toward safety and avoidance of errors tend to evaluate novel input as being less novel (Zhou et al., 2017). Hence, we expect that novel input disrupts idea generation for those high in need for structure, because the input is less closely related to their own mental images (Dugosh & Paulus, 2005) and existing knowledge, making it harder to assess and perceive the idea as being creative or to be stimulated by it (Dugosh & Paulus, 2005). Moreover, people high in need for structure are likely to have less positively valenced associations for novel ideas (cf. Zhou et al., 2017). We therefore expected that these people would not perceive novel input as a creative contribution (Gocłowska et al., 2014) and that this in turn would result in less cognitive stimulation than non-novel input.

## Theoretical Model

Our expectations are summarized in our theoretical model (see Figure 1). Novel input was expected to predict creativity perceptions, which in turn predicts cognitive stimulation: that is, productivity, idea generation, task enjoyment, and reduced feelings of being blocked. This indirect effect of novel input on cognitive stimulation was expected to be weakened by need for structure and strengthened by need for autonomy.

To test our propositions, we conducted an experiment where we assessed participants’ need strengths and manipulated the novelty of input. In Study 1, we tested our whole model, including the moderating role of both psychological needs and the mediating role of perceived creativity. In two additional studies, we examined whether participants might even prefer to receive no input at all rather than non-novel input (Study 2) or novel input (Study 3) that does not match their needs. We expected that people would show more favorable outcomes when not receiving any input than when receiving input mismatching their needs. Important to note is that Studies 2 and 3 included a “no input” control condition, so that it was not possible to test the mediating effect of perception of creativity of input in these studies. As all three experiments relied on the same method, we describe the combined methods below.

## Methods

### Samples and Design

Three laboratory studies were conducted to examine the causal relation between input novelty and brainstorming outcomes as moderated by the need for structure and autonomy (Studies 1-3) and mediated by the perceived creativity of input (Study 1). In these studies, participants brainstormed individually during a 10-min session on computers located in separate cubicles. However, all participants were led to believe they were working together with another participant via interactive online software.

#### Study 1

Participants were randomly assigned to one of two conditions (non-novel input [*n* = 39] vs. novel input [*n* = 39]). Seventy-eight undergraduate psychology students (36% male) voluntarily participated in this study for partial course credits. Their ages ranged between 18 and 24 years (*M* = 20.18, *SD* = 1.56).

#### Study 2

Participants were randomly assigned to one of two conditions (no input [*n* = 43] vs. novel input [*n* = 43]). Eighty-six undergraduate psychology students (42% male) voluntarily participated in this study for partial course credits. Their ages ranged between 19 and 29 years (*M* = 20.12, *SD* = 1.80).

#### Study 3

Participants were randomly assigned to one of two conditions (no input [*n* = 40] vs. non-novel input [*n* = 41]). Eighty-one students (33% male) of a Dutch university voluntarily participated in this study either for token payment (€5, approximately US$6.85) or for partial course credits. Their ages ranged between 18 and 29 years (*M* = 21.94, *SD* = 2.42). Most of the participants studied psychology (61%), followed by economics and business (16%), natural sciences (7%), law (7%), arts (6%), and medical sciences (3%).

### Procedure

Participants were seated at computers in individual cubicles. They were told that during this study they would “brainstorm together with another student via the Internet, to come up with ideas to create a healthy lifestyle.” In fact, however, all participants brainstormed individually. Before starting the brainstorm task, the participants filled out a questionnaire about their psychological need strengths, after which they were informed about the four brainstorming rules and were instructed to keep these in mind while brainstorming (see Osborn, 1957). The participants brainstormed for 10 min, after which they answered questions regarding the work process and their demographics.^1^ At the end of the study, the participants were thanked and debriefed.

#### Manipulation of input

For the experiments, we created an online brainstorming program, so as to enhance the idea of working together with another participant via the Internet. Also, participants who were in one of the input conditions were informed that they were able to exchange ideas with the other participant by pressing a “share” button and that the other participant could do the same. Because individuals typically generate about one idea per minute (Paulus, Larey, Putman, Leggett, & Roland, 2005), a total of nine preprogrammed pop-ups appeared, with intervals of 30, 60, or 90 s. The time intervals of these pop-ups were fixed but not constant, to avoid raising any suspicion about their preprogrammed nature. The pop-ups were said to display ideas shared by the other participant, but in fact showed preprogrammed ideas that had been previously rated by two independent experts in earlier unrelated research (Rietzschel et al., 2007) as either non-novel or novel, and as moderate on feasibility for all selected ideas.^2^ An example of non-novel input to increase health read “Don’t smoke,” and for novel input, “Add vitamins to chewing gum.” When a pop-up appeared, the idea presented was directly visible to the participant and had to be closed to be able to continue typing in ideas.

### Moderators and Mediator

Cronbach’s alphas of all variables are displayed in Tables 1, 5, and 7. Unless indicated otherwise, participants responded on a 5-point Likert-type scale ranging from 1 (*strongly disagree*) to 5 (*strongly agree*).

**Table 1.** Means, Standard Deviations, Correlations, and Cronbach’s Alphas Study 1 (*n* = 77).

Variable	*M*	*SD*	1	2	3	4	5	6	7	8	9	10
1. Sex (scored −1 for men, +1 for women)	NA	NA	NA									
2. Age	20.18	1.56	−.19^†^	NA								
3. Condition (scored −1 for non-novel input, +1 for novel input)	NA	NA	−.01	−.05	NA							
4. Need for structure	3.48	1.10	.20^†^	−.08	−.06	(.89)						
5. Need for autonomy	4.62	0.89	−.04	.01	−.12	.27*	(.83)					
6. Perceived creativity	3.35	1.19	.11	−.15	.61**	−.03	−.14	NA				
7. Productivity	10.32	3.14	−.03	.06	.02	−.03	−.06	.08	NA			
8. Idea diversity	6.49	1.85	−.03	.01	−.05	.12	−.09	.16	.65**	NA		
9. Task enjoyment	3.56	0.75	.08	−.04	−.13	−.17	−.07	.22^†^	.05	.13	(.87)	
10. Feeling blocked	2.83	1.12	.06	−.04	.01	.12	.01	.01	−.18	−.07	−.32**	NA

*Note*. When applicable, the corresponding Cronbach’s alpha is displayed on the diagonal.

† *p* < .10. *p < .05. **p < .01.

*Need for structure and need for autonomy* were each measured using four items of the Psychological Need Strength scale by Van Yperen, Rietzschel, and De Jonge (2014), which were adapted to fit the context of the current task. A sample item for need for structure is “In a brainstorming situation, I have a need for order and regularity”, and for need for autonomy, “In a brainstorming situation, I have a need to have a say in determining my activities and tasks.” Participants responded on a 7-point Likert-type scale ranging from 1 (*not at all*) to 7 (*to an extremely large extent*).

*Perceived creativity* (Study 1) was measured using one item: “The ideas I received from the other participant were creative.”

### Dependent Variables

*Productivity* was measured as the total number of non-duplicated ideas submitted per participant, that is, all ideas that did not directly overlap with previously stated ideas and were not identical to the preprogrammed input.

*Idea diversity* was defined as the number of different categories used, as independently coded by two trained raters who were blind to conditions. A category matrix system was used that crossed 12 specific goals (e.g., “improve bodily fitness”) with 10 means to reach these goals (e.g., “physical activity”), resulting in 120 different possible categories (see Nijstad et al., 2002). The second rater randomly rated 20% of these ideas. Agreement between the raters was high, with κ = .96 (95% confidence interval [CI] [.93, .99]), *p* < .0001), which we deemed sufficiently high to use the ratings of the first rater.

*Task enjoyment* was measured using four items from Van Yperen (2003), adapted to fit the current task. A sample item is “Did you enjoy doing the brainstorming task?”

*Feeling blocked* in coming up with new ideas during the brainstorming task was assessed using one item created for the purpose of this study: “I felt blocked in coming up with new ideas”.

## Results Study 1—Non-Novel Versus Novel Input

### Preliminary Analysis and Data Treatment

One participant in the novel input condition showed insufficient effort in responding (Huang, Curran, Keeney, Poposki, & DeShon, 2012).^3^ As inclusion of these data would likely lower the sample’s reliability, this participant was dropped from all analysis. Descriptives, correlations, and Cronbach’s alphas of all variables are given in Table 1. The highest correlations were obtained between productivity and idea diversity (*r* = .65, *p* < .001) and between condition and perceived creativity (*r* = .61, *p* < .001), the latter indicating that, as expected, participants on the whole perceived novel input as more creative than non-novel input (*M*_non-novel_ = 2.64 vs. *M*_novel_ = 4.08, *t*(75) = −6.64, *p* < .001). Also, a positive significant relation between need for structure and need for autonomy was found (*r* = .27, *p* = .02). To control for this relation in subsequent analyses, both need strengths were included simultaneously in the analyses of the moderated mediation model. Sex and age were evenly distributed across conditions, χ^2^_sex_(1, *N* = 78) = .00, *p* = 1.00; *F*_age_(1, 75) = .18, *p* = .67 (*M*_non-novel info_ = 20.26 vs. *M*_novel info_ = 20.11), and showed no significant effects on cognitive stimulation effects, *p*s_sex_ > .20 and *p*s_age_ > .25.^4^

### Hypothesis Testing

We used Hayes’ (2013) PROCESS SPSS macro (Model 58), with a bootstrapping sample size of 5,000, to test the conditional process model that input novelty would predict brainstorming outcomes through perceived creativity, and that this indirect path would be weakened by need for structure and strengthened by need for autonomy (see Figure 1). Following Hayes (2013), rather than conducting separate moderation and mediation analyses for parts of our model, we tested the total model in one analysis for each of the dependent variables.^5^

### Performance Component

#### Productivity

**Figure 2.**
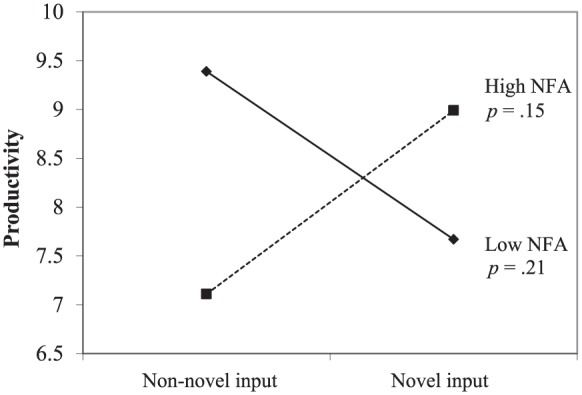
Study 1: Productivity as a function of input novelty and NFA. *Note.* NFA = need for autonomy.

In contrast to our expectations, no moderated mediation effects were obtained for productivity (see Table 2). We therefore investigated whether the direct effect of input novelty on productivity was moderated by need for structure and need for autonomy, without a mediating effect of perceived creativity. This regression analysis yielded a positive interaction of input novelty and need for autonomy, *b* = .91, *t*(69) = 2.10, *p* = .04, *R*^2^ = .08 (see Figure 2), but no significant interaction with need for structure (see Table 3). Simple slope analysis showed that novel input, as compared with non-novel input, was positively (but not significantly) associated with productivity when participants were high in need for autonomy, *b* = .76, *t*(69) *=* 1.47, *p* = .15, and negatively (but not significantly) associated with productivity when participants were low in need for autonomy, *b* = –.66, *t*(69) *=* –1.13, *p* = .21.

**Table 2.** Bootstrap Results for Moderated Mediation Study 1 (*n* = 77).

	Direct effect		Total model		Mediator effects			Moderator effects				
	Input novelty ➔ Outcome		Moderated mediation effect		Input novelty ➔ Perceived creativity		Perceived creativity ➔ Outcome		Input novelty × Need strength ➔ Perceived creativity		Perceived creativity × Need strength ➔ Outcome	
	*b* value (*SE*)	95% CI	*b* value (*SE*)	95% CI	*b* value (*SE*)	95% CI	*b* value (*SE*)	95% CI	*b* value (*SE*)	95% CI	*b* value (*SE*)	95% CI
Need for structure												
Productivity	−0.15 (0.47)	[−1.09, 0.79]	0.22 (0.29)	[−0.35, 0.79]	1.29** (0.38)	[0.52, 2.05]	−0.01 (1.19)	[−2.38, 2.35]	−0.17 (0.10)	[−0.37, 0.04]	0.09 (0.33)	[−0.56, 0.75]
Idea diversity	**0.27**^†^ (0.27)	[−0.98, 0.08]	**0.35** (0.17)	[0.06, 0.71]	1.29** (0.38)	[0.52, 2.05]	0.21 (0.67)	[−1.12, 1.55]	−0.17 (0.10)	[−0.37, 0.04]	0.08 (0.18)	[−0.29, 0.45]
Task enjoyment	−0.31** (0.10)	[−0.52, −0.11]	**0.21** (0.07)	[0.09, 0.38]	1.29** (0.38)	[0.52, 2.05]	0.15 (0.26)	[−0.36, 0.66]	−0.17 (0.10)	[−0.37, 0.04]	0.04 (0.07)	[−0.10, 0.18]
Feeling blocked	0.02 (0.17)	[−0.32, 0.35]	−0.01 (0.12)	[0.25, 0.22]	1.29** (0.38)	[0.52, 2.05]	0.27 (0.42)	[−0.57, 1.11]	−0.17 (0.10)	[−0.37, 0.04]	−0.08 (0.12)	[−0.31, 0.15]
Need for autonomy												
Productivity	−0.19 (0.47)	[−1.12, 0.74]	0.17 (0.28)	[−0.41, 0.70]	−0.30 (0.59)	[−1.48, 0.88]	−1.67 (1.65)	[−4.96, 1.61]	**0.22**^†^ (0.13)	[−0.03, 0.47]	0.41 (0.33)	[−0.25, 1.08]
Idea diversity	**−0.46**^†^ (0.27)	[−0.99, 0.07]	**0.33** (0.16)	[0.05, 0.71]	−0.30 (0.59)	[−1.48, 0.88]	0.06 (0.94)	[−1.82, 1.93]	**0.22**^†^ (0.13)	[−0.03, 0.47]	0.09 (0.19)	[−0.29, 0.47]
Task enjoyment	−0.32** (0.10)	[−0.52, −0.12]	**0.20** (0.07)	[0.08, 0.37]	−0.30 (0.59)	[−1.48, 0.88]	0.03 (0.36)	[−0.68, 0.74]	**0.22**^†^ (0.13)	[−0.03, 0.47]	0.06 (0.07)	[−0.09, 0.20]
Feeling blocked	0.05 (0.16)	[−0.27, 0.37]	0.03 (0.11)	[−0.19, 0.23]	−0.30 (0.59)	[−1.48, 0.88]	**1.39** (0.57)	[0.26, 2.52]	**0.22**^†^ (0.13)	[−0.03, 0.47]	−0.29* (0.11)	[−0.52, −0.07]

*Note*. If CI does not include zero, the effect is considered statistically significant and is displayed in bold. CI = confidence interval.

† *p* < .10. *p < .05. **p < .01. ***p < .001.

**Table 3.** Results for the Moderated Regression Analysis Study 1 (*n* = 77).

Regression model	Productivity	
	*b* value (*SE*)	95% CI
Intercept	7.91 (4.86)	[−1.78, 17.60]
Sex	−0.09 (0.40)	[−0.89, 0.72]
Age	0.12 (0.24)	[−0.35, 0.60]
Condition	0.05 (0.37)	[−0.68, 0.78]
Need for structure	0.01 (0.36)	[−0.70, 0.73]
Need for autonomy	−0.25 (0.43)	[−1.12, 0.62]
Condition × Need for structure	−0.40 (0.36)	[−1.12, 0.31]
Condition × Need for autonomy	0.91* (0.43)	[0.04, 1.76]
*R* ^2^	.27	
Adjusted *R*^2^	.07	

*Note.* Unstandardized regression coefficients are shown. CI = confidence interval.

† *p* < .10. *p < .05. **p < .01. ***p < .001.

#### Idea diversity

**Figure 3.**
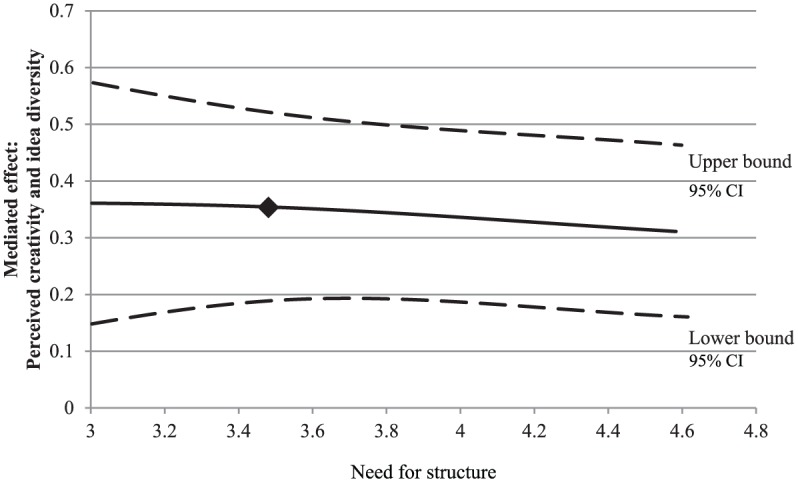
Study 1: A plot of the conditional indirect effect of input novelty on idea diversity through perceived creativity, conditioned on the moderator (need for structure), with 95% confidence bands. *Note.* The square indicates the mean level of the need strength. CI = confidence interval.

**Figure 4.**
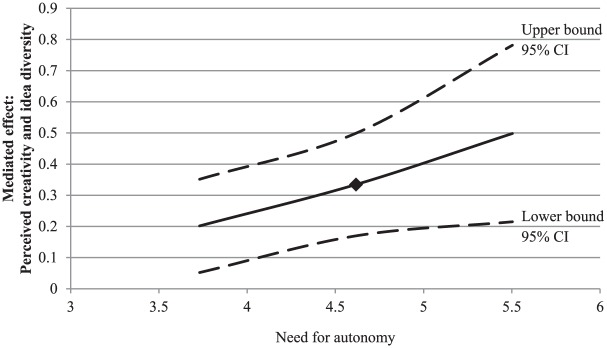
Study 1: A plot of the conditional indirect effect of input novelty on idea diversity through perceived creativity, conditioned on the moderator (need for autonomy), with 95% confidence bands. *Note.* The square indicates the mean level of the need strength. CI = confidence interval.

As expected, the conditional indirect effect of input novelty on idea diversity through perceived creativity was significant for both need strengths (need for structure: *b* = .35, 95% CI [.06, .71], need for autonomy: *b* = .33, 95% CI [.05, .71]; see also Table 2).^6^ On the whole, people who received novel input rather than non-novel input were more flexible in their generation of ideas, as the conditional effects at low, moderate, and high levels were all positive (see Table 4). However, in line with our hypotheses, this effect was weaker for participants with higher levels of need for structure and for participants with lower levels of need for autonomy. As can be seen in Figure 3, the positive effect of input novelty on idea diversity through perceived creativity was weaker for people high in need for structure than for those low in need for structure. Conversely, the positive effect of novelty on idea diversity through perceived creativity was stronger for people high in need for autonomy than for people low in need for autonomy (see Figure 4).

**Table 4.** Bootstrap Results for Moderated Mediation at Different Levels of the Moderator Study 1 (*n* = 77).

	Value need strength	Idea diversity		Task enjoyment		Feeling blocked	
		*b* value (*SE*)	95% CI	*b* value (*SE*)	95% CI	*b* value (*SE*)	95% CI
Need for structure							
Low	2.38	0.36 (0.29)	[−0.14, 1.02]	**0.22** (0.13)	[0.08, 0.52]	0.06 (0.19)	[−0.34, 0.40]
Moderate	3.48	**0.35** (0.17)	[0.06, 0.71]	**0.21** (0.07)	[0.09, 0.38]	−0.01 (0.12)	[−0.25, 0.22]
High	4.58	**0.31** (0.15)	[0.08, 0.72]	**0.18** (0.07)	[0.08, 0.36]	−0.06 (0.12)	[−0.33, 0.17]
Need for autonomy							
Low	3.73	0.20 (0.15)	[−0.03, 0.59]	**0.12** (0.08)	[0.01, 0.32]	0.15 (0.10)	[−0.00, 0.43]
Moderate	4.62	**0.33** (0.16)	[0.05, 0.71]	**0.20** (0.07)	[0.08, 0.37]	0.03 (0.11)	[−0.19, 0.23]
High	5.50	**0.50** (0.28)	[0.00, 1.10]	**0.30** (0.12)	[0.10, 0.56]	−0.20 (0.17)	[−0.56, 0.12]

*Note*. Low, moderate, and high levels of the need strengths are constituted as the M-level of the need strength, ± 1 *SD*. If CI does not include zero, the moderated mediation effect is considered statistically significant and is displayed in bold. CI = confidence interval.

### Psychological Component

#### Task enjoyment

**Figure 5.**
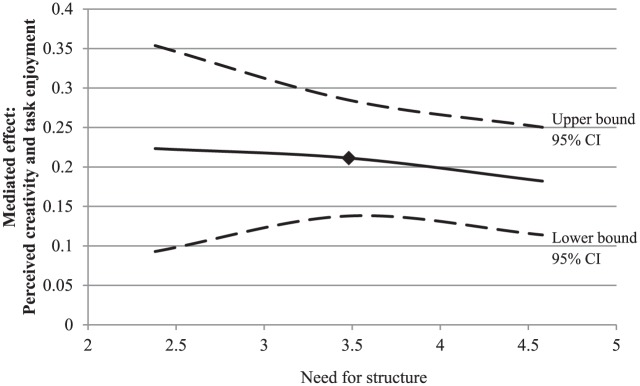
Study 1: A plot of the conditional indirect effect of input novelty on task enjoyment through perceived creativity, conditioned on the moderator (need for structure), with 95% confidence bands. *Note.* The square indicates the mean level of the need strength. CI = confidence interval.

**Figure 6.**
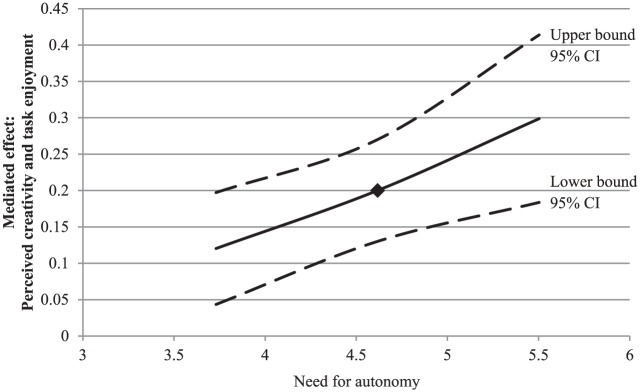
Study 1: A plot of the conditional indirect effect of input novelty on task enjoyment through perceived creativity, conditioned on the moderator (need for autonomy), with 95% confidence bands. *Note.* The square indicates the mean level of the need strength. CI = confidence interval.

As expected, the conditional indirect effect of input novelty on task enjoyment through perceived creativity was significantly moderated by both needs (need for structure: *b* = .21, 95% CI [.09, .38], need for autonomy: *b* = .20, 95% CI [.08, .37]; see Note 6). On the whole, people who received novel input rather than non-novel input enjoyed the task more, as the conditional effects at low, moderate, and high levels were all positive (see Table 4). However, in line with our hypotheses, this effect was weaker for participants with higher levels of need for structure, and for participants with lower levels of need for autonomy. As can be seen in Figure 5, the positive effect of input novelty on task enjoyment through perceived creativity was weaker for people high in need for structure than for those low in need for structure. Conversely, the positive effect of novelty on task enjoyment through perceived creativity was stronger for people high in need for autonomy than for people low in need for autonomy (see Figure 6).

#### Feeling blocked

**Figure 7.**
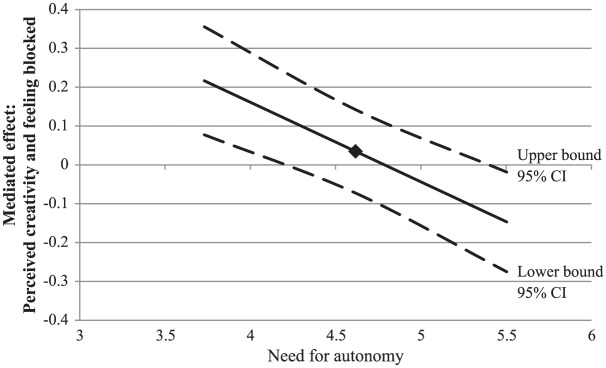
Study 1: A plot of the conditional indirect effect of input novelty on feeling blocked through perceived creativity, conditioned on the moderator (need for autonomy), with 95% confidence bands. *Note.* The square indicates the mean level of the need strength. CI = confidence interval.

In contrast to what was expected, no conditional indirect effect of input novelty on feeling blocked was obtained (see Table 2). However, when we focused on the need for autonomy, we found two separate moderation effects in the model that were in line with our expectations. These indicated a positive interaction effect for input novelty and need for autonomy on perceived creativity (i.e., for the first part of the model), and a negative interaction effect for perceived creativity and need for autonomy on feeling blocked (i.e., for the second part of the model; see Table 2). As can be seen in Figure 7, the positive effect of input novelty on feeling blocked through perceived creativity was weaker for people high in need for autonomy than for people low in need for autonomy. No effects were obtained for need for structure.

## Discussion: Study 1

The results of Study 1 indicate that novel input indeed has an indirect effect on cognitive stimulation through perceived creativity. As expected, this path was moderated by psychological needs, such that those with a high (vs. low) need for structure and those with a low (vs. high) need for autonomy benefited less from exposure to novel ideas. The type of input that results in cognitive stimulation apparently is not the same for everybody, and ideas intended to be helpful are in fact not always cognitively stimulating. In such instances where a misfit is created, it may be better not to receive any ideas at all. We conducted two additional experiments to test this question. In these studies, participants either received input or not, with the type of input (novel or non-novel) differing between the two studies. For participants with a high need for structure, we expected more cognitive stimulation when receiving no input than when receiving highly novel input (Study 2). For participants with a high need for autonomy, we expected more cognitive stimulation when receiving no input than when receiving non-novel input (Study 3).

## Results Study 2—Novel Input and Need for Structure

### Preliminary Analyses and Data Treatment

Descriptives, correlations, and Cronbach’s alphas for all variables are given in Table 5. Similarly to Study 1, the highest correlation was obtained for productivity and idea diversity (*r* = .84, *p* < .001). The relation between need for structure and need for autonomy (*r* = .26, *p* = .02) was taken into account by creating regression models that included both moderators. Sex and age were more or less evenly distributed across conditions, χ^2^_sex_(1, *N* = 86) = .76, *p* = .38, and *F*_age_(1, 84) = .71, *p* = .40 (*M*_novel info_ = 20.28 vs. *M*_no info_ = 19.95).

**Table 5.** Means, Standard Deviations, Correlations, and Cronbach’s Alphas Study 2 (*n* = 86).

Variable	*M*	*SD*	1	2	3	4	5	6	7	8	9
1. Sex (scored −1 for men, +1 for women)	NA	NA	NA								
2. Age	20.12	1.79	−.13	NA							
3. Condition (scored −1 for no input, +1 for novel input)	NA	NA	.09	−.09	NA						
4. Need for structure	3.56	1.00	.25*	.12	.03	(.89)					
5. Need for autonomy	4.51	0.90	.12	−.01	.10	.26*	(.83)				
6. Productivity	9.64	4.25	−.12	−.19^†^	−.11	−.03	−.03	NA			
7. Idea diversity	5.93	2.23	−.25*	−.13	−.09	−.08	.01	.84**	NA		
8. Task enjoyment	3.44	0.77	.12	.05	.16	.11	−.18	.17	.18	(.88)	
9. Feeling blocked	2.92	1.20	.10	−.09	.22*	.04	.15	−.18^†^	−.18^†^	−.14	NA

*Note*. When applicable, the corresponding Cronbach’s alpha is displayed on the diagonal.

† *p* < .10. *p < .05. **p < .01.

### Hypothesis Testing

For all dependent variables, hypotheses were tested by running a regression analysis with input, need for structure, need for autonomy, and the two interaction terms of input with the needs. To represent the interaction between input (dummy coded −1 = no input, 1 = input) and psychological needs, the need variable under investigation was first standardized and then multiplied by condition (Aiken & West, 1991). Last, sex (with two levels, “−1” for men and “1” for women) and age were included as covariates in all analyses and indicated no significant effects on brainstorming outcomes: *p*s > .15 for sex and *p*s > .10 for age, with some exceptions (see Table 6; Note 4).

**Table 6.** Results for the Moderated Regression Analyses Study 2 (*n* = 86).

Regression model	Dependent variables							
	Productivity		Idea diversity		Task enjoyment		Feeling blocked	
	*b* value^a^	95% CI	*b* value^a^	95% CI	*b* value^a^	95% CI	*b* value^a^	95% CI
Intercept	20.01***	[9.45, 30.75]	10.29***	[4.73, 15.86]	2.61**	[0.74, 4.48]	4.15**	[1.20, 7.11]
Sex	−0.69	[−0.17, 0.28]	−0.62*	[−1.12, −0.11]	0.11	[−0.06, 0.28]	0.05	[−0.22, 0.32]
Age	−0.51^†^	[−1.04, 0.02]	−0.21	[−0.49, 0.06]	0.04	[−0.05, 0.13]	−0.06	[−0.21, 0.08]
Condition	−0.47	[−1.38, 0.45]	−0.20	[−0.67, 0.29]	0.13	[−0.03, 0.29]	0.23^†^	[−0.02, 0.49]
Need for structure	0.03	[−0.96, 1.01]	−0.03	[−0.55, 0.48]	0.12	[−0.05, 0.30]	−0.02	[−0.29, 0.26]
Need for autonomy	−0.06	[−1.14, 1.03]	0.11	[−0.46, 0.68]	−0.25*	[−0.44, −0.06]	0.23	[−0.07, 0.53]
Condition × Need for structure	0.14	[−0.83, 1.10]	0.00	[−0.51, 0.51]	−0.20*	[−0.37, −0.03]	0.26^†^	[−0.01, 0.53]
Condition × Need for autonomy	−0.81	[−1.89, 0.28]	−0.20	[−0.76, 0.37]	0.07	[−0.12, 0.26]	0.04	[−0.27, 0.34]
*R* ^2^	.10		.11		.16		.13	
Adjusted *R*^2^	.01		.02		.08		.05	

*Note.* CI = confidence interval.

a Unstandardized regression coefficients are shown.

† *p* < .10. *p < .05. **p < .01. ***p < .001.

### Performance Component

#### Productivity

Contrary to expectations, no main or interaction effects were obtained.

#### Idea diversity

Contrary to expectations, only a negative main effect for sex was obtained (*b =* −.62, *t* = −2.42, *p* = .02).

### Psychological Component

#### Task enjoyment

**Figure 8.**
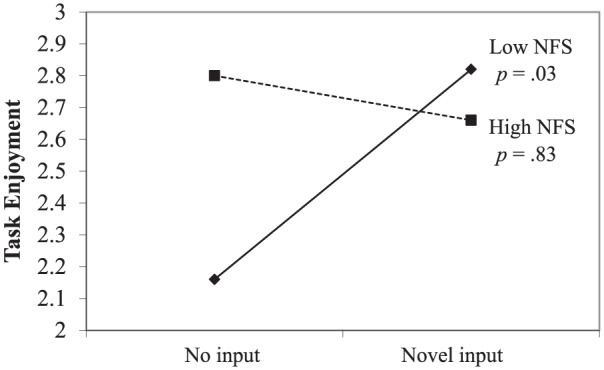
Study 2: Task enjoyment as a function of no input versus novel input and NFS. *Note.* NFS = need for structure.

In line with hypotheses, the regression analysis yielded a negative interaction effect of input and need for structure, *b* = −.20, *t*(78) = −2.31, *p* = .02 (see Figure 8). However, contrary to expectations, simple slopes analysis showed that novel input, as compared with no input, resulted in higher levels of task enjoyment for participants low in need for structure (*b* = .26, *t* = 2.16, *p* = .03), but did not significantly affect participants high in need for structure (*b* = −.03, *t* = −.21, *p* = .83). The regression analysis also revealed a negative main effect for need for autonomy, indicating that those high in need for autonomy enjoyed the task less, *b* = −.25, *t*(78) = −2.59, *p* = .01.

#### Feeling blocked

**Figure 9.**
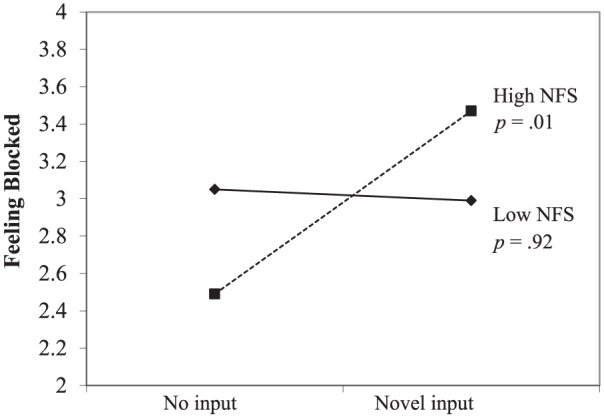
Study 2: Feeling blocked as a function of no input versus novel input and NFS. *Note.* NFS = need for structure.

As expected, the regression analysis yielded a positive interaction effect of input and need for structure, *b* = .26, *t*(78) = 1.94, *p* = .057 (see Figure 9). Simple slopes analysis showed that novel input, as compared with no input, was positively (and significantly) associated with feeling blocked when participants were high in need for structure (*b =* .48, *t* = 2.67, *p* = .01), but not when participants were low in need for structure (*b =* .02, *t* = 1.00, *p* = .92).

## Results Study 3—Non-Novel Input and Need for Autonomy

### Preliminary Analyses and Data Treatment

Descriptives and Cronbach’s alphas of all variables are given in Table 7. The highest correlation was obtained between productivity and idea diversity (*r* = .85, *p* < .001). The relation between need for structure and need for autonomy (*r* = .33, *p* < .001) was taken into account by creating regression models that included both moderators. Sex and age were more or less evenly distributed across conditions, χ^2^_sex_(1, *N* = 81) = 1.21, *p* = .27, and *F*_age_(1, 79) = .10, *p* = .75 (*M*_no input_ = 21.85 vs. *M*_non-novel input_ = 22.02), and indicated no significant effects on brainstorming outcomes, *p*s > .15 for sex and *p*s > .10 for age, with some exceptions (see Table 8; Note 4).

**Table 7.** Means, Standard Deviations, Correlations, and Cronbach’s Alphas Study 3 (*n* = 81).

Variable	*M*	*SD*	1	2	3	4	5	6	7	8	9
1. Sex (scored −1 for men, +1 for women)	NA	NA	NA								
2. Age	21.94	2.42	−.32**	NA							
3. Condition (scored −1 for no input, +1 for non-novel input)	NA	NA	.12	−.04	NA						
4. Need for structure	3.38	1.15	.35**	−.29**	.04	(.87)					
5. Need for autonomy	4.51	1.06	.12	.07	.04	.33**	(.88)				
6. Productivity	9.86	4.94	.08	−.05	.01	−.04	−.04	NA			
7. Idea diversity	6.41	2.70	.28*	−.08	.25*	.06	.05	.85**	NA		
8. Task enjoyment	3.85	0.75	.19	.19	.11	.15	.31**	−.04	.02	(.88)	
9. Feeling blocked	2.53	1.16	.21	−.16	.08	.24*	−.08	−.05	.13	−.24*	NA

*Note*. When applicable, the corresponding Cronbach’s alphas are displayed on the diagonal.

† *p* < .10. *p < .05. **p < .01.

**Table 8.** Results for the Moderated Regression Analyses Study 3 (*n* = 81).

Regression model	Dependent variables							
	Productivity		Idea diversity		Task enjoyment		Feeling blocked	
	*b* value^a^	95% CI	*b* value^a^	95% CI	*b* value^a^	95% CI	*b* value^a^	95% CI
Intercept	11.63*	[0.05, 23.20]	6.21*	[0.27, 12.15]	1.93*	[0.36, 3.51]	2.89*	[0.36, 5.41]
Sex	0.58	[−0.78, 1.95]	0.83*	[0.13, 1.53]	0.13	[−0.05, 0.32]	0.16	[−0.14, 0.46]
Age	−0.09	[−0.61, 0.43]	−0.00	[−0.27, 0.26]	0.09*	[0.02, 0.16]	−0.02	[−0.13, 0.09]
Condition	0.00	[−1.14, 1.15]	0.57^†^	[−0.02, 1.15]	0.06	[−0.10, 0.22]	0.07	[−0.18, 0.32]
Need for structure	−0.37	[−1.55, 0.82]	−0.16	[−0.77, 0.44]	0.08	[−0.09, 0.24]	0.21	[−0.05, 0.46]
Need for autonomy	−0.12	[−1.30, 1.06]	0.04	[−0.56, 0.65]	0.19*	[0.03, 0.35]	−0.18	[−0.44, 0.08]
Condition × Need for structure	0.31	[−0.79, 1.42]	0.21	[−0.36, 0.77]	−0.11	[−0.26, 0.04]	−0.06	[−0.30, 0.18]
Condition × Need for autonomy	−0.34	[−1.50, 0.82]	−0.05	[−0.65, 0.54]	−0.02	[−0.18, 0.13]	0.27*	[0.01, 0.52]
*R* ^2^	.02		.14		.21		.16	
Adjusted *R*^2^	−.07		.05		.14		.08	

*Note.* CI = confidence interval.

a Unstandardized regression coefficients are shown.

† *p* < .10. *p < .05. **p < .01. ***p < .001.

### Hypothesis Testing

For all dependent variables, hypotheses were tested by running regression analyses similar to those in Study 2. All regressions are summarized in Table 8.

### Performance Component

#### Productivity

Contrary to expectations, no main or interaction effects were obtained.

#### Idea diversity

Contrary to expectations, only a positive main effect for sex was obtained, *b* = .83, *t*(75) = 2.37,*p* = .02.

### Psychological Component

#### Task enjoyment

Contrary to expectations, only a positive main effect for age and need for autonomy was obtained, *b* = .09, *t*(75) = 2.41, *p* = .02 and *b* = .19, *t*(75) = 2.37, *p* = .02, respectively.

#### Feeling blocked

**Figure 10.**
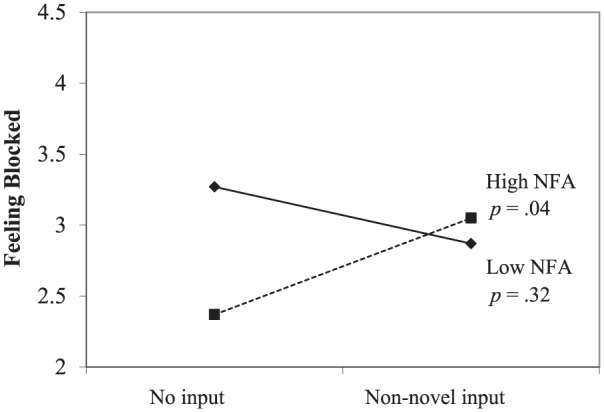
Study 3: Feeling blocked as a function of no input versus non-novel input and NFA. *Note.* NFA = need for autonomy.

As expected, the regression analysis yielded a positive interaction of input and need for autonomy, *b* = .27, *t*(75) = 2.10, *p* = .04 (see Figure 10). Simple slopes analysis showed that non-novel input (relative to no input) resulted in feeling blocked when participants were high in need for autonomy (*b =* .37, *t* = 2.06, *p* = .04), but not when participants were low in need for autonomy (*b =* −.13, *t* = −1.01, *p* = .32).

## General Discussion

In the present research, we aimed to address the role of novelty and individual differences in cognitive stimulation during brainstorming. We expected and found that the indirect effect of input novelty on cognitive stimulation through perceived creativity is *weakened* by the need for structure and *strengthened* by the need for autonomy. Specifically, people high in need for structure did not perceive highly novel ideas as creative and therefore showed lower idea diversity and less task enjoyment. In addition, receiving novel input (compared with not receiving input) resulted in a tendency to enjoy the task somewhat (although not significantly) less, and in feeling more blocked in generating ideas. These results are in line with the notion that participants high in need for structure do not appreciate novel input that adds complexity and ambiguity, but prefer non-novel input that provides them with clarity, predictability, and certainty.

We found more or less the opposite pattern for participants high in need for autonomy: They perceived novel input as more creative, which in turn predicted higher idea diversity and task enjoyment, as well as lower feelings of being blocked. In addition, receiving novel input resulted in higher productivity for them relative to non-novel input, regardless of their perception of the input as being creative. Also, people high in need for autonomy felt more blocked in their ideation process when they received non-novel input than when they did not receive input at all. These results are in line with the notion that participants high in need for autonomy do not appreciate non-novel input that does not have an added information value over and above the obvious; however, novel input is welcomed as a useful and creative contribution that will help them brainstorming. In contrast to our expectation, no performance-related outcomes were obtained in Studies 2 and 3; we provide a possible explanation below (see “Limitations and Future Directions”).

### Implications

It seems that people can benefit from the ideas of others (Brown et al., 1998; Dugosh et al., 2000; Stroebe, Nijstad, & Rietzschel, 2010), but only if the input received fits their psychological needs and is positively perceived as a creative contribution. In practice, organizations and teams could benefit from our findings by taking both components into account. First, being aware of the individual needs of team members rather than using a “one size fits all” approach would be important when aiming to increase productivity and cognitive stimulation. Managers or teams could, for example, discuss the needs and preferences with the employees and could use the short need strength scale (Van Yperen et al., 2014) as a basis for this conversation. Electronic brainstorming could be used as a tool to adapt the brainstorm setting to one’s personal needs.

Second, the positive perception of another’s idea as being creative depends on its stimulating potential (Zhou et al., 2017). This perception differs per individual, but perceiving the input as creative seems crucial for its potential to further stimulate the creative process. It may therefore be fruitful to train people to reflect on all types of input as having a creative potential. People high in need for structure could, for example, be taught how to deal with and use original or unusual input (e.g., as a useful tool to consider a problem from a new angle), whereas people high in need for autonomy could be made aware of the potential benefits of receiving less original input (e.g., as a starting point to generate more original ideas themselves). Training teams to value information diversity might be a useful starting point in this regard, in order to stimulate the active consideration of the viewpoints and ideas of others. Previous research has indicated that such positive diversity beliefs increase the performance of informationally diverse groups, as it helps people to elaborate more on the information shared (Homan et al., 2007). This enhanced elaboration of information may increase one’s positive perception of the input as being creative, thereby increase the number of associated ideas people generate based on the input. Future research is needed to investigate whether these expectations indeed hold.

### Limitations and Future Directions

While the findings of the current studies may already be useful for group brainstorming, a possible direction for future creativity research would be to further investigate the effect of perceived creativity and of individual needs. First, more research could investigate the mediating role of perceived creativity in cognitive stimulation, as its role could only be investigated in Study 1. The perception of input as being creative seems to relate to its stimulating potential (Zhou et al., 2017), which makes it interesting to investigate the underlying process of how specific ideas from others stimulate the generation of additional ideas. Cognitive stimulation is normally assessed at a global, interpersonal level (e.g., differences in productivity), but it should be possible to also study it on the level of ideas or strings of ideas within participants. Creating such a measure could provide more insight as to which aspects in presented ideas have stimulating effects and how people continue brainstorming from this input. As previous research indicates that ideas are appreciated as a creative contribution when these activate new associations in one’s mind (Zhou et al., 2017), one could expect that this type of input activates overlapping cognitive responses or associations. This could result in clustering or persistence if people stay within the same category as the stimulus item, or alternatively, could result in flexibility if people combine their currently activated mental category with the category of the input to generate further ideas.^7^

Second, more research is needed on the role that individual needs play in cognitive stimulation. Perhaps whether brainstorming input is helpful also depends on the fit of these ideas with one’s currently activated mental schemas, especially when people have a high need for structure. For them, diverse input that activates new mental categories may work disruptively, as this input requires additional information processing and does not fit with their current structure. This line of reasoning fits previous work showing that schema-inconsistent information (Gocłowska et al., 2014) and socially distant information that reinforces new modes of thinking (Baer, 2010) can increase or decrease creative performance, depending on one’s needs. Related to this, cognitive diversity in groups seems to work better for some people than for others, especially for those who score high on agreeableness, extraversion, or openness to experience (Nakui, Paulus, & van der Zee, 2011). Similarly, people high in need for autonomy may perceive cognitive diversity as a welcome addition for group brainstorming as it increases the chance of receiving more diverse ideas that could result in new or novel insights. In contrast, those high in need for structure may experience cognitive diversity in the group as unwelcome, as diverse insights and ideas from others further increase complexity and ambiguity in the task.

Third, it would be interesting to examine the process behind the (mis)fit of brainstorming input and psychological needs. In contrast to our expectations, Studies 2 and 3 showed significant effects of input-needs fit only on the psychological component of cognitive stimulation, and not on the performance component. We can only speculate as to why this is the case. It is possible that participants’ emotional responses to the task played a role here. For example, a “misfit” situation may have caused participants to feel angry or frustrated. Activating emotions, whether positive (such as enthusiasm) or negative (such as anger and fear), can stimulate creativity (Nijstad, De Dreu, Rietzschel, & Baas, 2010; Yang & Hung, 2015). Hence, the expected drop in performance in the “misfit” conditions in Studies 2 and 3 may have been counteracted by the positive effect of these activating emotions. Other work suggests also that experiencing anger when receiving mismatching external input may be expected for those high in need for autonomy. The functional goal resulting from anger is to regain freedom in one’s actions and to remove external control (Yang & Hung, 2015), an end state that is typically desired by those high in need for autonomy. For Study 1, in which we compared a fit versus misfit situation, we would speculate that both conditions activated emotions, the first positive and the latter negative ones, hence canceling each other out. Further research could include measures of emotions to test this reasoning and to unravel the effects of mismatching input on participants’ emotions. Related to this, it would be interesting to include a measure of memory for the presented ideas, to get an indication as to whether individual differences also affect the extent to which participants pay attention to the ideas presented to them. For example, it may be that those high in need for autonomy pay less attention to the presented ideas, as ideas of others mismatch their preference to work on their own. In turn, this could result in less associational impact from the input (see Note 7).

Finally, in the present studies we focused on individual differences in need for structure and need for autonomy. Although these needs are important predictors and moderators in the context of creative performance, work motivation, and group interactions (Chirumbolo et al., 2004; Deci & Ryan, 2000; Van Yperen et al., 2016), it would be interesting to also address the role of other individual differences, such as mood, processing mode, openness to experience, extraversion-introversion, and approach and avoidance temperament (Baas, Roskes, Sligte, Nijstad, & De Dreu, 2013; Baer, 2010; Jung, Lee, & Karsten, 2012; Nijstad et al., 2010). Mapping the ways in which various individual differences moderate cognitive stimulation effects may also help us understand the underlying mechanisms and identify further boundary conditions for stimulation to occur.

## Conclusion

Creative performance is highly valued and necessary to achieve innovative behavior and organizational effectiveness (Amabile, 1983; Paulus & Nijstad, 2003). Given that group work is ubiquitous in modern organizations and that group brainstorming remains highly popular despite the risks of productivity loss, it is important to understand more about the factors that contribute to (or inhibit) the psychological and performance component of cognitive stimulation. The current findings add to our understanding by showing that the level of cognitive stimulation depends on input novelty, perceptions of creativity, and people’s psychological needs. There is a need for more research on creativity, focused on the role of psychological needs, in order to better understand the mechanisms through which creative performance unfolds, and to be able to create the ideal circumstances for people to experience cognitive stimulation when brainstorming.

## Supplementary Material

Supplementary material

## Notes

1. Exploratively, additional variables were included concerning individual needs, task perception, and performance.

2. For the novel condition, ideas were selected that were rated ≥4 (on a 5-point scale) on novelty, for the non-novel condition, ideas were selected that were rated ≤2 on novelty, *t*(16) = −13.91, *p* < .0001 (*M*_non-novel input_ = 1.56 vs. *M*_novel input_ = 4.00). Feasibility was held constant at a moderate level in both conditions, with an average of 3.25 on a 5-point scale, *t*(16) = −1.47, *p* = .16 (*M*_non-novel input_ = 3.00 vs. *M*_novel input_ = 3.56).

3. This was manifested by the response of “strongly agree” to all items, including original and reversed items. In addition, the participant indicated not to have responded carefully to the questions and that we should not use the data.

4. Analyzing the data without including covariates led to a similar pattern of results.

5. The PROCESS analysis gives insight in the complete moderated mediation model (Hayes, 2013). For the curious reader, we analyzed the interaction between novelty and need for structure (need for autonomy) predicting perceived creativity (thus, only analyzing the first part of the model). This resulted in significant interactions that are in line with what is expected: a negative interaction effect for need for structure, *b* = −.22, *t*(69) = −2.06, *p* = .04, and a positive interaction effect for need for autonomy, *b* = .27, *t*(69) = 2.17, *p* = .03.

6. Investigating the specific paths revealed that the direct effect of input novelty on cognitive stimulation was non-significant for both models and that the single moderation effects were not uniquely significant (see Table 2). Only the complete moderated mediation models could explain our findings.

7. We thank an anonymous reviewer for pointing this out.
