# Supplementary material for: Stimulated by Novelty? The Role of Psychological Needs and Perceived Creativity
Source: Pers Soc Psychol Bull. 2018 Feb 6;44(6):851–67. doi: 10.1177/0146167217752361 (PMC5946663; doi:10.1177/0146167217752361)
Supplement: Supplementary material [file de_jonge_online_appendix.pdf]

The Psychological Need Strength scale (Van Yperen et al., 2013), adapted to fit the current task. Measured on a 7-point Likert scale:

1 = not at all
2 = to a very small extent
3 = to a small extent
4 = to a moderate extent
5 = to a large extent
6 = to a very large extent
7 = to an extremely large extent

During this study, you will be working on a brainstorming task. The following questions are about your needs when working in such a situation.

Need for autonomy
In a brainstorming situation, I have the need…

…. to have a say in determining my activities and tasks.
…. to decide on my own how to go about getting my job done.
…. to determine on my own how to best approach my work.
…. for freedom to do my work in the way that I think is best.

Need for structure
In a brainstorming situation, I have the need…

…. for order and regularity.
…. for rules and guidelines that I can follow.
…. to know exactly what to expect.
…. for a daily routine.

Task instruction when receiving non-novel or novel input.

Task instruction: please read this carefully!

As you may know, the city of Groningen attracts many students. However, living a student life increases stress and unhealthy behavior for many students. With this study, we try to find as many ways as possible in which living a healthy life can be improved - for students, but also for people in general.

In order to help you to come up with ideas for a healthy Groningen, we will use a brainstorming technique. You will brainstorm online together with another person who is participating from the Facuty of Arts at the RUG.

The topic on which you'll be brainstorming is: **How can healthy living in Groningen be improved?**

Your task, together with the other person, is to come up with as many ideas, solutions, or suggestions as you can think of for a healthy Groningen.

Please keep the following rules in mind during this task:

1. The more ideas, the better.
2. The more unusual the idea, the better.
3. Combine and improve produced ideas.
4. Don't criticize ideas produced by yourself and the other.

Write down all ideas that come to mind, no matter how wild. Try to be concise and to the point in the phrasing of your ideas.

During this task, you'll be able to send and receive ideas from each other. To share an idea, simply press the 'share' button shown next to the idea you submitted. When the other participant shares an idea with you, a pop-up screen will appear, presenting the idea. Please read the ideas provided by the other participant carefully and use these to come up with new ideas.

You and the other student will have 10 minutes to work on this task. After 10 minutes, you will automatically be send to the next page.
Go to the next page to start the brainstorming task.

The brainstorming task was presented for 10 minutes.

Task instruction when not receiving any input.

Task instruction: please read this carefully!

As you may know, the city of Groningen attracts many students. However, living a student life increases stress and unhealthy behavior for many students. With this study, we try to find as many ways as possible in which living a healthy life can be improved - for students, but also for people in general.

In order to help you to come up with ideas for a healthy Groningen, we will use a brainstorming technique. You will brainstorm online together with another person who is participating from the Facuty of Arts at the RUG.

The topic on which you'll be brainstorming is: **How can healthy living in Groningen be improved?**

Your task, together with the other person, is to come up with as many ideas, solutions, or suggestions as you can think of for a healthy Groningen.

Please keep the following rules in mind during this task:

1. The more ideas, the better.
2. The more unusual the idea, the better.
3. Combine and improve produced ideas.
4. Don't criticize ideas produced by yourself and the other.

Write down all ideas that come to mind, no matter how wild. Try to be concise and to the point in the phrasing of your ideas.

You and the other student will have 10 minutes to work on this task. After 10 minutes, you will automatically be send to the next page.
Go to the next page to start the brainstorming task.

The brainstorming task was presented for 10 minutes.

Outcome variables, all measured on a 5-point Likert scale:

1 = strongly disagree
2 = disagree
3 = neither agree nor disagree
4 = agree
5 = strongly agree

Task Enjoyment Scale from Van Yperen (2003), adapted to fit the current task.

Did you enjoy doing the brainstorm task?
Did you take interest in doing the brainstorm task?
Are you interested in doing tasks like this?
Did you feel pleasant while you were doing the brainstorm task?

Feeling blocked measure

I felt blocked to come up with new ideas.

Perceived creativity measure

The ideas I received from the other participant were creative.
